# Co-exposure to lead and cadmium is associated with increased severity of social deficits in children with autism spectrum disorders

**DOI:** 10.3389/fnut.2026.1678007

**Published:** 2026-01-29

**Authors:** Hongjie Mao, Yifang Qian, Yanling Su, Xiumei Liu, Xiuzhen Nie, Yingying Cai

**Affiliations:** Fujian Children's Hospital, Developmental and Behavioral Pediatrics, Fuzhou, China

**Keywords:** autism spectrum disorders, cadmium, lead, mineral, vitamin

## Abstract

**Objective:**

This case–control study aimed to compare micronutrient (vitamins and minerals) profiles between children with autism spectrum disorders (ASD) and age- and gender-matched typically developing (TD) controls. It further sought to explore the associations of these profiles with core symptom severity and neurodevelopmental outcomes in ASD.

**Methods:**

We enrolled 50 children with ASD and 50 matched TD controls. Core ASD symptoms were assessed using the Autism Diagnostic Observation Schedule-Second Edition (ADOS-2) and Childhood Autism Rating Scale (CARS), while neurodevelopmental status was evaluated with the Gesell Developmental Scale (GDS). Serum vitamin and mineral levels were measured in all participants. Eating behaviors were assessed using the Preschooler’s Eating Behavior Questionnaire (PEBQ).

**Results:**

Key findings included: (1) significantly more severe eating behavior disturbances in ASD children, characterized by increased food selectivity, reduced self-feeding ability, and maladaptive eating patterns; (2) markedly higher serum iron levels in the ASD group (*p* = 0.028); (3) elevated serum lead (Pb) and cadmium (Cd) levels were positively correlated with social communication impairments. Notably, a combined heavy metal exposure index (reflecting Pb-Cd co-exposure) remained significantly associated with these impairments after controlling for screen time; (4) higher serum levels of vitamins D and B12 were associated with better gross motor development, whereas increased copper (Cu) levels were inversely associated with motor function. Elevated calcium (Ca) levels were positively associated with adaptive behavior development.

**Conclusion:**

These results demonstrate that both nutritional imbalances and co-exposure to heavy metals such as lead and cadmium are significantly associated with behavioral symptoms and neurodevelopmental outcomes in ASD. The findings underscore the importance of integrating routine nutritional surveillance and assessment of environmental heavy metal exposure to guide personalized interventions in this population. These cross-sectional associations warrant further investigation in larger, longitudinal studies that incorporate detailed dietary and environmental assessments to explore potential causal mechanisms.

## 1 Introduction

Autism spectrum disorder (ASD) represents a complex array of neurodevelopmental conditions characterized by significant clinical heterogeneity (1, 2). Core manifestations include impaired social communication, restricted interests, and repetitive behaviors, with symptom severity existing along a broad continuum (3, 4). Recent epidemiological data reveal a rising global prevalence, currently affecting 1 in 36 children aged 8 years in the United States and 0.7–1.0% of pediatric populations in China. Notably, ASD demonstrates a marked male predominance (male-to-female ratio ≈4:1) (5). The underlying pathophysiology of ASD remains incompletely elucidated, and the condition imposes considerable personal, familial, and societal burdens due to its lifelong nature and substantial care requirements.

Beyond the hallmark neurodevelopmental impairments, children with ASD frequently present with significant comorbidities, including marked eating behavior disturbances and gastrointestinal dysfunction (6). Growing evidence indicates that children with ASD exhibit substantially higher rates of food selectivity and feeding difficulties than their neurotypical peers. Estimates suggest that approximately “46–92%” of children with ASD may experience such issues, compared to a much lower rate among typically developing (TD) children (7). These challenges are thought to stem from the core features of ASD, such as sensory processing differences and behavioral inflexibility, as well as from a high prevalence of co-occurring psychiatric and somatic conditions. These comorbidities, which include anxiety and gastrointestinal disorders, can further disrupt appetite, metabolism, and eating patterns (8). These aberrant eating behaviors, in turn, may contribute to or coexist with a higher prevalence of metabolic abnormalities in this population.

Essential micronutrients serve critical functions in neurodevelopment and the maintenance of neurological homeostasis (9, 10). The current body of research presents conflicting evidence regarding nutritional status in ASD. Numerous domestic and international studies have demonstrated significantly different serum levels of key nutrients in children with ASD compared to neurotypical controls. For example, the research indicates that vitamin D (VD) deficiency is markedly prevalent in ASD, affecting approximately 67.7% of affected children compared to much lower rates in the general population (11). Similarly, iron deficiency has been reported in 24.1–83.3% of children with ASD, significantly higher than community baselines (12, 13). These nutritional deficiencies have been associated with more pronounced social communication deficits and increased repetitive behaviors. Importantly, clinical trials have suggested that supplementation with VD and folic acid may ameliorate certain ASD symptoms (4, 14, 15). However, conflicting evidence exists in the literature. Several well-designed studies (16, 17) found no significant differences in these serum biomarkers between ASD and control groups. Notably, Liu et al. (18) reported no meaningful correlations between symptom severity and concentrations of ferritin, folic acid, VD, or vitamin B12 (VB12). Similar inconsistencies have been observed in studies investigating other micronutrients, including vitamins A, E, K, B12, and minerals such as zinc (Zn), copper (Cu), magnesium (Mg), and calcium (Ca), with prevalence estimates for imbalances varying widely across studies (19, 20).

These discrepancies may stem from methodological variations, sample size limitations, ethnic differences, or environmental confounding factors. The current body of research regarding nutritional biomarkers in ASD remains inconclusive. Given the high prevalence of multiple potential micronutrient imbalances and their theorized link to core symptoms, our study systematically measured a comprehensive panel of these vitamins and minerals to clarify their profile in our cohort.

Beyond isolated nutritional deficiencies or excesses, the overall metabolic milieu and neurodevelopmental trajectory in ASD may be critically shaped by the interplay between nutrient status and exposure to environmental neurotoxicants. Essential nutrients and toxic metals often share absorption pathways and biological targets. For instance, deficiencies in minerals such as iron, zinc, and calcium can increase the intestinal absorption and tissue accumulation of divalent heavy metals like lead (Pb) and cadmium (Cd) (21), while also impairing the body’s antioxidant defenses against metal-induced oxidative stress. Conversely, these toxic metals can disrupt the homeostasis of essential nutrients, creating a vicious cycle that may exacerbate neurodevelopmental impairment. Environmental exposure to neurotoxic heavy metals is a widespread concern. Biomonitoring studies show detectable levels of lead and cadmium in a significant proportion of children globally, with evidence suggesting that even low-level exposure may contribute to neurodevelopmental risks (22). Therefore, a comprehensive assessment requires the simultaneous evaluation of both nutritional and toxic elemental profiles. Exposure to environmental neurotoxicants, particularly heavy metals like lead (23, 24) and cadmium (25), has been hypothesized to be associated with ASD etiology and symptom severity (26). Study findings indicated elevated blood levels of lead (27%) and cadmium (22%) in children with ASD relative to healthy controls (27). These metals are pervasive environmental contaminants known to disrupt neurodevelopmental processes, including those involved in social cognition and communication. To investigate this interplay and the potential independent contribution of toxicant exposure, we quantitatively measured serum Pb and cadmium Cd concentrations using high-precision methods.

In the context of our study conducted in Fuzhou, potential local sources of lead and cadmium exposure for children may include historical industrial legacy (e.g., from past electronics manufacturing or battery production), ambient dust in urban areas, and potential contamination from certain locally produced foods or traditional products. Their inclusion in our analysis is justified by epidemiological associations between metal exposure and ASD risk, as well as mechanistic evidence linking them to oxidative stress, synaptic dysfunction, and neurotransmitter imbalances (23)—pathways implicated in social deficit domains of ASD. Importantly, most prior investigations relied on parent-reported questionnaires for symptom assessment, which lack the sensitivity to discriminate between distinct symptom domains (e.g., social deficits versus stereotyped behaviors). This limitation is particularly problematic given the substantial phenotypic heterogeneity within the ASD population. To address these methodological concerns, our study employs the Autism Diagnostic Observation Schedule-Second Edition (ADOS-2), the gold-standard observational assessment for ASD. By systematically analyzing the relationships between serum nutrient and toxic metal profiles and core symptom domains (including social impairment, communication deficits, and restricted/repetitive behaviors), we aim to provide novel insights into ASD pathophysiology and inform evidence-based nutritional interventions.

## 2 Materials and methods

### 2.1 Study design and participants

This hospital-based cross-sectional study enrolled 50 drug-naïve children with ASD (aged 2–10 years) diagnosed at Fujian Children’s Hospital’s Department of Developmental and Behavioral Pediatrics (January 2024–January 2025). Diagnosis was confirmed by two board-certified pediatric neurologists using DSM-5 criteria.

### 2.2 Exclusion criteria

Children were excluded if they had:

1. Comorbid neurodevelopmental disorders (e.g., intellectual disability, ADHD).
2. Active neurological/psychiatric conditions.
3. Known genetic/metabolic disorders.
4. Chronic inflammatory/autoimmune diseases.
5. Recent infections (<4 weeks) or antibiotic use.
6. Special diets (e.g., ketogenic, gluten-free) within 3 months.
7. Micronutrient supplements exceeding 150% RDA within 3 months.

### 2.3 Control group selection

Fifty age- and sex-matched typically developing children (2–10 years) were recruited from the well-child clinic at the Department of Developmental and Behavioral Pediatrics, Fujian Children’s Hospital, during the same study period (January 2024 to January 2025). Inclusion required:

1. Normal developmental screening (ASQ-3 score ≥ cutoff in all domains).
2. No medical conditions affecting vitamin metabolism (e.g., liver/kidney disease).
3. No acute/chronic infections within 12 weeks before enrollment.
4. No high-dose micronutrient supplementation (>150% RDA) within 12 weeks.

### 2.4 Ethical considerations and data collection

This voluntary study obtained written informed consent from all participants’ legal guardians before enrollment and was approved by the Institutional Review Board of Fujian Children’s Hospital (Ethics Approval No. 2024ETKLR08003). Trained research staff conducted structured face-to-face interviews with caregivers of both ASD and TD children using standardized questionnaires. Collected data included:

1. Demographic characteristics (child’s age, gender, ethnicity, family socioeconomic status, parental education level). We also collected data on potential confounding factors, including maternal parity, caregiver age, and education level.
2. Comprehensive medical history.
3. Eating behaviors assessed by the Preschooler’s Eating Behavior Questionnaire (PEBQ).

The Autism Diagnostic Observation Schedule-Second Edition (ADOS-2) and the Childhood Autism Rating Scale (CARS) were used to assess the child’s core symptoms. The ADOS-2 (28) is a semi-structured, standardized assessment of communication, social interaction, play, and restricted and repetitive behaviors, in which the child’s behaviors are observed in a standardized activity situation by a professional assessor. Five modules are included: modules 1 through 3 provide a comparative score indicating the level of autism spectrum-related symptoms compared to children with autism who are the same age and have similar language skills. The GESELL Developmental Scale (GDS) was used to assess the child’s level of neurodevelopment. The Preschooler’s Eating Behavior Questionnaire (PEBQ) was used to assess eating behavior problems in preschoolers. Dietary intake was assessed via caregiver report using the PEBQ. While this method does not provide quantitative nutrient data, it is a validated tool for characterizing eating behavior patterns and food selectivity in young children, which was the focus of our behavioral assessment.

### 2.5 Biochemical measurements

All children underwent fasting blood collection at the Laboratory of Fujian Provincial Children’s Hospital. Serum levels of vitamins (A, D, E, K, B12), ferritin, and essential minerals were measured using standard clinical laboratory methods (including chemiluminescence immunoassay and colorimetric assays). Specifically, serum levels of the heavy metals lead and cadmium were quantitatively analyzed using inductively coupled plasma mass spectrometry (ICP-MS) (29), a gold-standard method for trace metal detection due to its high sensitivity and specificity. All analyses were performed by the laboratory technicians of Fujian Children’s Hospital.

### 2.6 Statistical analysis

SPSS 25.0 software was used for statistical analysis. (1) The use of normality tests (Shapiro–Wilk) to guide test selection; (2) Clear criteria for using the Student’s t-test (for normal data) vs. the Mann–Whitney U-test (for non-normal data); (3) A description of the hierarchical regression approach used to control for confounding (screen time) and address multicollinearity (via a combined Heavy Metal Index). A normality test was performed on all data. Results are expressed as means ± standard deviations (SD) or median (25th, 75th percentiles) for continuous variables and percentages for categorical variables. To compare levels between groups, the two-tailed Student’s t-test, the chi-square test, and the Mann–Whitney U-test were used. To determine the significance of the variables of interest, Spearman’s correlation study was performed. To address potential confounding and multicollinearity, hierarchical regression analyses were conducted. Specifically, due to high collinearity between serum lead and cadmium levels (Variance Inflation Factor, VIF > 47), a combined ‘Heavy Metal Exposure Index’ was created by summing the z-scores of both metals. This index was then used in hierarchical models to examine its association with clinical outcomes after controlling for screen time as a covariate. All comparisons were made using a two-sided test with a significance threshold of 0.05. *p* < 0.05 was considered a statistically significant difference.

## 3 Result

### 3.1 Demographic characteristics

The study population consisted of 50 children diagnosed with ASD and 50 age- and sex-matched TD children. The ASD group showed a male-to-female ratio of approximately 2.1:1 (68% male, 32% female). Comparative analysis revealed no statistically significant differences between the two groups in gender distribution, family type, daily screentime, allergy history, delivery mode, maternal low mood during pregnancy, or prenatal folic acid supplementation (all *p* > 0.05). However, significant differences were observed in maternal reproductive history, with the number of pregnancies and parity both showing statistically significant variations (*p* < 0.05 for both). Additionally, primary caregivers of children with ASD were significantly older and had lower educational attainment compared to caregivers of TD children. It is important to note that the primary focus of this study was to investigate the relationships between serum biomarkers (micronutrients and heavy metals) and clinical outcomes. While the aforementioned sociodemographic and perinatal differences represent potential confounding variables, our core analytical strategy was to examine biomarker-symptom associations within the ASD group, thereby controlling for broad between-group differences. These findings suggest potential associations between maternal reproductive factors and ASD risk, while also highlighting socioeconomic differences in caregiver characteristics between the two groups, as detailed in Table 1.

**Table 1:** Demographic characteristics of ASD and TD children.

Parameter	Category	ASD children (*n* = 50)	TD children (*n* = 50)	z/t/X2	*p*
Age (year), Median (IQR)		5.92(4.40, 7.08)	5.25(4.15, 7.48)	−0.503	0.615
Sex	Male (%)	34(68)	28(56)	1.528	0.216
	Female (%)	16(32)	22(42)		
Family type	Core family (%)	16(32)	24(48)	2.667	0.102
	Extended family (%)	34(68)	26(52)		
Screen time(h)	<1	12(24)	12(24)	1.317	0.518
	1–2	17(34)	22(44)		
	>2	21(42)	16(32)		
Number of pregnancies (%)	1	15(30)	30(60)	10.712	0.005^*^
	2	22(44)	16(32)		
	≥3	13(26)	4(8)		
Number of births (%)	1	19(38)	35(0.7)	11.007	0.004^*^
	2	26(52)	14(28)		
	≥3	5(10)	1(2)		
Delivery mode	Vaginal delivery (%)	30(60)	35(70)	1.099	0.295
	Cesarean section (%)	20(40)	15(30)		
Maternal low mood during pregnancy		23(46)	30(60)	1.967	0.161
Prenatal folic acid supplementation		44(88)	46(92)	0.444	0.505
Have siblings		34(68)	15(30)	14.446	0.000*
Mothers’ full-time foster care		28(56)	30(60)	0.164	0.685
Child’s primary caregiver	Parents	36(72)	32(64)	0.735	0.391
	Outer/grandparents	14(28)	18(36)		
Age of primary caregiver Median (IQR)		37(32, 53)	34(30, 57)	−1.288	0.198
Educational qualifications of the main breadwinner	Middle school and below	26(52)	17(34)	4.484	0.214
	High School/Middle School	12(24)	18(36)		
	Three-year college	8(0.16)	7(14)		
	Undergraduate and above	4(8)	8(16)		
Allergy history		16(32)	10(20)	1.871	0.171

ASD, autism spectrum disorder; TD, typically developing; Data are expressed as means ± SD or median (25th, 75th percentiles) or percentages for categorical variables. IQR, interquartile range; ^*^p < 0.05.

### 3.2 Comparison of eating behaviors of children in the ASD and TD groups

The analysis revealed statistically significant differences in eating behaviors between children with ASD and TD controls. Specifically, children with ASD exhibited greater food selectivity (picky eating), more maladaptive eating habits, higher prevalence of exogenous eating patterns, and reduced self-feeding ability compared to their TD peers(all *p* < 0.05). These findings indicate that eating behavior disturbances are more pronounced in ASD and may significantly impact their nutritional status and overall health. Detailed results are presented in Table 2.

**Table 2:** Eating behaviors between ASD and TD children.

Variable	ASD	TD	t/z	*p*
Picky eating, mean ± SD	3.00 ± 0.79	1.97 ± 0.51	7.106	0.000^*^
Food response, mean ± SD	2.84 ± 0.97	2.59 ± 0.37	1.546	0.128
Maladaptive eating habits, median (IQR)	2.6(2.0，2.8)	2.0(1.8，2.2)	−4.091	0.000^*^
Satiety responsiveness, mean ± SD	2.54 ± 0.73	2.28 ± 0.44	1.929	0.058
Exogenous eating patterns, mean ± SD	1.97 ± 0.41	2.26 ± 0.57	−2.689	0.009^*^
Emotional eating	1.4(1.0，2.2)	1.9(1.5，2.0)	−1.912	0.056
Self-feeding ability, median (IQR)	2.0(1.8，2.1)	3.1(2.6，4.0)	−6.233	0.000^*^

Data are expressed as means ± SD or median (25th, 75th percentiles) for eating behaviors. IQR, interquartile range. ^*^p < 0.05.

### 3.3 Comparison of nutrient and mineral levels between the ASD and TD groups

Given the more pronounced eating behavior disturbances observed in children with ASD, we analyzed serum concentrations of key nutrients, including vitamins (A, D, E, K, B12) and minerals (Zn, Fe, Ca, Mg, Cd, and Pb). As shown in Table 3, children with ASD exhibited significantly higher serum iron levels compared to TD controls (*p* < 0.05). However, no statistically significant differences were found between the two groups for the remaining nutrients analyzed (vitamins A, D, E, K, B12; Zn, Ca, Mg, Cd, and Pb). These findings suggest that while iron deficiency may be a particular nutritional concern in ASD, other micronutrient levels appear comparable to typically developing children.

**Table 3:** Vitamins and minerals in ASD and TD children.

Variant	ASD	TD	t/z	*p*
VA	0.35(0.26, 0.46)	0.34(0.28, 0.38)	−0.984	0.325
VD	25.08 ± 10.82	25.19 ± 8.14	−0.057	0.995
VE	8.57 ± 2.38	8.87 ± 2.25	−0.635	0.527
VK	0.67(0.26, 1.33)	0.56(0.25, 0.94)	−1.065	0.128
VB12	899.00(574.25,1335.50)	906.00(781.55, 1180.50)	−1.442	0.106
Fe	7.92(7.63, 8.55)	7.82(7.44, 8.39)	−1.299	0.028*
Zn	69.81 ± 8.92	67.73 ± 9.53	1.129	0.262
Cu	17.13 ± 1.90	16.91 ± 2.21	0.547	0.585
Mg	1.71(1.61, 1.77)	1.66(1.55, 1.77)	−1.231	0.218
Ca	1.78(1.71, 1.88)	1.78(1.67, 1.85)	−0.038	0.970
Cd	0.32(0.29, 0.38)	0.35(0.30, 0.39)	−1.263	0.206
Pb	27.28(24.23, 32.01,)	29.15(24.11, 33.75)	−1.275	0.202

VA, vitamin A; VD, vitamin D; VE, vitamin E; VK, vitamin K; VB12, vitamin B12; Fe, iron; Zn, zinc; Cu, copper; Mg, magnesium; Ca, calcium; Cd, cadmium; Pb, lead; Data are expressed as means ± SD or median (25th, 75th percentiles). ^*^p < 0.05.

### 3.4 Correlation of micronutrients with clinical symptom severity in children with ASD

**Figure 1:**
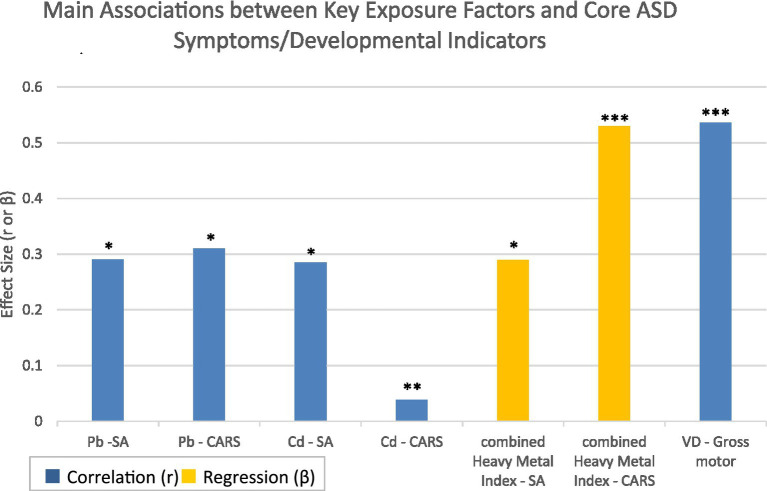
Main associations between key exposure factors and core ASD symptoms/developmental indicators. Bar chart displaying standardized beta coefficients (*β*) from regression analyses (for the combined heavy metal index) and Spearman’s correlation coefficients (*r*) for individual biomarkers. Positive values indicate that higher biomarker levels are associated with higher scores (more severe symptoms or better development). *p < 0.05, **p < 0.01, ***p < 0.001. ASD, autism spectrum disorder; SA, social ffect score; CARS, Childhood Autism Rating Scale.

**Figure 2:**
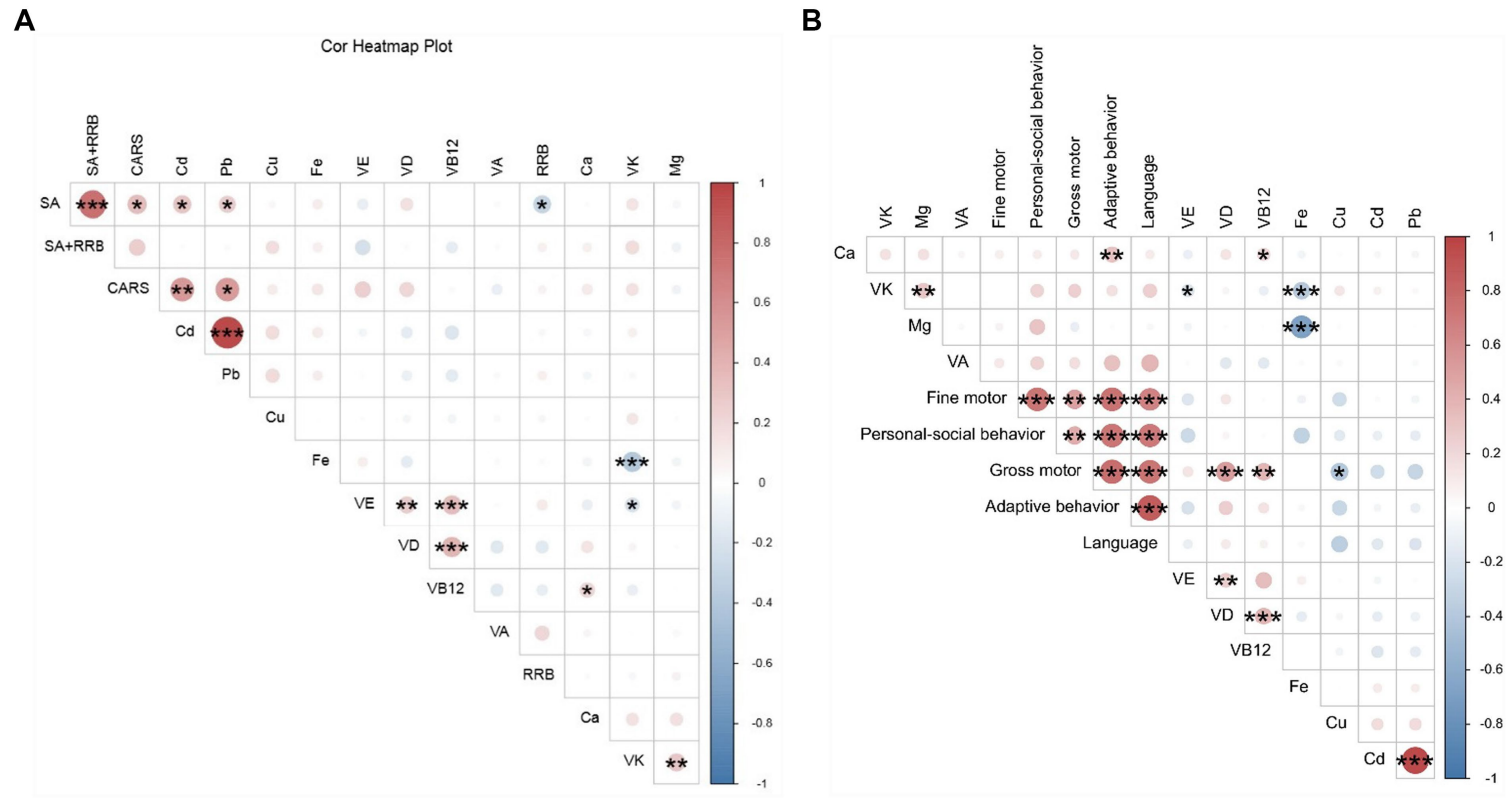
**(A)** Correlation matrix between a comprehensive set of serum biomarkers and core autism symptom domains. Heatmap depicting Spearman’s correlation coefficients (*ρ*) for associations between serum levels of vitamins (A, D, E, K, B12), minerals/essential elements (Fe, Cu, Mg, Ca), heavy metals (Pb, Cd), and core ASD symptom severity scores (CARS total, SA, RRB, SA + RRB) within the ASD group. Each cell color and numerical value represents the correlation coefficient, with red indicating a positive correlation and blue a negative correlation. Color intensity corresponds to the magnitude of the correlation. Statistical significance was assessed via two-tailed tests, with *p < 0.05, **p < 0.01, ***p < 0.001. **(B)** Correlation matrix between serum biomarkers and neurodevelopmental outcomes across functional domains. Heatmap illustrating Spearman’s correlation coefficients (ρ) for associations between the same panel of serum biomarkers and developmental quotient scores from the Gesell Developmental Schedules (GDS) within the ASD group. The GDS domains include: adaptive, gross motor, fine motor, language, and personal-social. The color scale and interpretation follow those in statistical significance: *p < 0.05, **p < 0.01, ***p < 0.01. GDS, Gesell Developmental Schedules.

This study investigated the relationship between heavy metal exposure and autism symptom severity using the CARS and the ADOS-2. As shown in Table 4, Comprehensive analysis revealed statistically significant positive correlations between Pb levels and both CARS total scores (*r* = 0.310, *p* = 0.028) and ADOS-2 Social Affect (SA) domain scores (*r* = 0.291, *p* = 0.041). Similarly, the serum Cd levels showed significant positive associations with CARS total scores (*r* = 0.378, *p* = 0.007) and ADOS-2 SA scores (*r* = 0.285, *p* = 0.045). Notably, neither heavy metal demonstrated significant correlations with restricted/repetitive behavior (RRB) scores. For a visual summary of the key effect sizes from these analyses, see Figure 1. Furthermore, the complete correlation matrix between all serum biomarkers and core symptom domains is presented in Figure 2A. These consistent findings across multiple assessment measures strongly suggest that environmental exposure to lead and cadmium may collectively contribute to more severe social communication impairments in children with autism spectrum disorder.

**Table 4:** Correlation between micronutrient levels and severity of clinical symptoms in children with ASD.

Variable	CARS		SA		RRB		SA + RRB	
	*r*	*p*	*r*	*p*	*r*	*p*	*r*	*p*
VA	−0.105	0.468	0.053	0.714	0.040	0.783	0.052	0.719
VD	0.201	0.162	0.118	0.416	−0.042	0.774	−0.083	0.566
VE	0.157	0.275	−0.019	0.896	0.095	0.511	−0.011	0.938
VK	0.157	0.276	0.068	0.639	−0.020	0.892	−0.047	0.747
VB12	−0.743	0.767	−0.053	0.718	0.061	0.679	−0.088	0.547
Fe	0.126	0.384	0.206	0.152	0.002	0.900	0.206	0.152
Zn	0.074	0.610	0.014	0.332	−0.062	0.667	0.297	0.360
Cu	0.066	0.650	−0.025	0.864	0.059	0.683	0.204	0.154
Mg	0.067	0.646	0.052	0.718	0.129	0.370	0.121	0.403
Ca	0.117	0.419	−0.111	0.444	0.082	0.573	−0.034	0.812
Cd	0.378	0.007^*^	0.285	0.045^*^	−0.228	0.511	0.171	0.236
Pb	0.310	0.028^*^	0.291	0.041^*^	−0.201	0.161	0.157	0.277

*p < 0.05; CARS, the Childhood Autism Rating Scale; SA, Social Affect; RRB, restricted/repetitive behavior. The potential confounding effect of screen time was assessed. Screen time was not correlated with social deficit scores (both *p* > 0.8). After controlling for screen time in hierarchical regression, a combined Heavy Metal Index (sum of z-scored Pb and Cd) remained significantly associated with both CARS (*β* = 0.530, *p* < 0.001) and SA scores (*β* = 0.290, *p* = 0.044).

To assess the potential confounding role of screen time, we first examined its association with core social deficit measures within the ASD group. Screen time was not significantly correlated with either the CARS total score (*r* = 0.010, *p* = 0.946) or the SA score (*r* = −0.029, *p* = 0.842), indicating it is not a linear predictor of social impairment severity in our sample. However, to statistically control for this variable, and to address the significant multicollinearity between serum lead and cadmium (Variance Inflation Factor, VIF > 47), we constructed a combined Heavy Metal Exposure Index (sum of z-scores for Pb and Cd). Hierarchical regression analyses were then performed. After adjusting for screen time in the first step, the Heavy Metal Index remained a significant predictor of more severe symptoms in the second step: for the CARS total score, standardized *β* = 0.530, *p* < 0.001; for the SA score, standardized *β* = 0.290, *p* = 0.044. Screen time itself did not contribute significantly to either model (both *p* > 0.7).

### 3.5 Association between micronutrient levels and neurodevelopmental outcomes in children with ASD

GDS were employed to evaluate neurodevelopmental status in children with ASD, with lower developmental quotient (DQ) scores indicating greater developmental delay. As shown in Table 5, higher serum levels of vitamins D and B12 were associated with better gross motor development, whereas increased Cu levels were inversely associated with motor function (all *p* < 0.05). Additionally, serum Ca concentrations showed a positive association with adaptive behavior scores. However, no significant correlations were observed between other measured micronutrients and developmental domains. The overall pattern of associations between serum biomarkers and neurodevelopmental outcomes is visualized in the correlation matrix shown in Figure 2B. These findings suggest that specific micronutrients may selectively influence distinct neurodevelopmental trajectories in children with ASD.

**Table 5:** Correlation between developmental levels and micronutrients in children with ASD.

Variable	Gross motor		Fine motor		Adaptive behavior		Language		Personal-social behavior	
	*r*	*p*	*r*	*p*	*r*	*p*	*r*	*p*	*r*	*p*
VA	0.168	0.319	0.036	0.833	0.132	0.438	0.149	0.377	0.024	0.889
VD	0.536	0.001^*^	0.160	0.345	0.232	0.167	0.141	0.404	0.104	0.542
VE	0.192	0.255	−0.099	0.560	−0.179	0.288	−0.076	0.056	−0.275	0.100
VK	0.233	0.165	−0.099	0.561	0.146	0.389	0.065	0.704	0.005	0.978
VB12	0.476	0.003^*^	0.013	0.550	0.238	0.162	0.194	0.257	0.103	0.549
Fe	−0.157	0.352	−0.023	0.894	0.001	0.995	−0.048	0.780	−0.132	0.437
Zn	−0.283	0.090	−0.158	0.351	−0.101	0.550	−0.138	0.416	0.013	0.983
Cu	−0.401	0.014^*^	−0.298	0.373	−0.195	0.249	−0.211	0.210	−0.068	0.689
Mg	0.015	0.930	0.099	0.559	0.047	0.781	0.050	0.768	0.043	0.801
Ca	0.143	0.399	0.122	0.473	0.423	0.009^*^	0.106	0.533	0.138	0.415
Cd	−0.210	0.211	−0.069	0.683	0.020	0.908	−0.100	0.557	−0.094	0.579
Pb	−0.277	0.097	−0.115	0.499	−0.046	0.787	−0.090	0.596	−0.126	0.456

*p < 0.05.

## 4 Discussion

This study primarily investigated the association between micronutrient and mineral status in children with ASD and core symptom severity. Four major findings emerged from our research: (1) significantly more severe eating behavior disturbances in ASD children, characterized by increased food selectivity, reduced self-feeding ability, and maladaptive eating patterns; (2) markedly higher serum iron levels in the ASD group (*p* = 0.028); (3) elevated serum Pb and Cd levels were positively correlated with social communication impairments. Notably, a combined heavy metal exposure index (reflecting Pb-Cd co-exposure) remained significantly associated with these impairments after controlling for screen time; (4) higher serum levels of vitamins D and B12 were associated with better gross motor development, whereas increased Cu levels were inversely associated with motor function. Elevated Ca levels were positively associated with adaptive behavior development. These results collectively highlight the importance of nutritional factors and heavy metal exposure in both behavioral manifestations and neurodevelopmental progression in ASD.

Emerging evidence consistently identifies eating behavior disturbances as one of the most prevalent comorbidities in children with ASD (30, 31), with considerable heterogeneity in the manifestation and severity of these feeding challenges across clinical studies. Characteristic difficulties encompass problematic mealtime behaviors, selective food refusal, markedly restricted food preferences, insistence on specific utensils or food presentation formats, and preferential consumption of pureed or low-texture foods (6, 32, 33). Our findings corroborate this established evidence by demonstrating significantly greater severity of eating-related impairments in children with ASD compared to their typically developing peers, particularly evident in heightened food selectivity, compromised self-feeding abilities, and maladaptive eating patterns. These co-occurring feeding difficulties likely arise from complex interactions between core ASD features. These include stereotypic behaviors that may precipitate ritualized eating habits, social communication deficits that impair the acquisition of normative feeding skills, and cognitive delays that may contribute to difficulties in mastering self-feeding techniques. This complexity highlights the critical need for developmentally informed interventions.

Our study revealed elevated serum iron levels in children with ASD compared to typically developing controls. This finding appears to contrast with some prior reports of iron deficiency in this population (34, 35) yet aligns with other evidence suggesting complex iron dyshomeostasis in ASD (36). The discrepancy may stem from heterogeneity in ASD cohorts, differences in dietary patterns, or the use of iron supplementation. In our sample, the observed elevation could be attributable to dietary preferences favoring iron-rich foods (e.g., fried meats) over vegetables (37), coupled with potential widespread use of nutritional supplements. The relationship between iron status and ASD symptoms is likely non-linear and multifactorial. While severe iron deficiency can disrupt neurodevelopment and dopaminergic signaling, potentially affecting language and cognition (38–41), iron overload may also be detrimental, promoting neuroinflammation and oxidative stress (42). Our data, showing elevated levels but not a linear correlation with symptom severity within the ASD group, support this notion of a complex “dual risk” profile rather than a simple deficiency model. Therefore, we advocate for a precision medicine approach: routine monitoring of iron status (serum ferritin and iron) should be integrated into the clinical management of children with ASD. Supplementation should be initiated only upon confirmation of deficiency, as indiscriminate supplementation in replete individuals could theoretically exacerbate risks. Future longitudinal studies are needed to delineate the causal pathways linking iron homeostasis, diet, supplementation practices, and ASD symptomatology.

Converging evidence implicates environmental heavy metal exposure, particularly to Pb and Cd, in the pathophysiology of ASD (43–45). Our study adds to this body of work by demonstrating significant positive correlations between blood concentrations of both Pb and Cd and the severity of core social deficits in children with ASD. It is important to note that the observed correlation coefficients, while statistically significant, are modest in magnitude. This suggests that heavy metal levels, while associated with symptom severity, are likely part of a multifactorial etiology rather than sole determinants. The neurotoxic mechanisms of Pb are well-documented, including disruption of calcium signaling, neurotransmitter systems (e.g., dopamine, GABA), synaptic plasticity, and induction of oxidative stress and neuroinflammation (46–48). Cd may contribute through pro-inflammatory pathways (e.g., Th17 cell differentiation) and by disrupting zinc-dependent antioxidant defenses (49, 50). Importantly, recent perspectives highlight that neurotoxicity extends beyond neurons to include glial cell dysfunction, which is crucial for metal homeostasis and neuroinflammation, providing a broader framework for understanding these effects (51).

A key methodological insight from our data is the high collinearity between Pb and Cd levels, indicating that co-exposure is the normative pattern. Therefore, the associations observed in our study between heavy metal exposure and social deficits were independent of screen time, a potential behavioral confounder, as confirmed by our supplementary analyses. Furthermore, the significant multicollinearity between lead and cadmium levels in our cohort suggests that co-exposure, rather than isolated effects, is the prevailing exposure pattern. To address this, we constructed a combined Heavy Metal Exposure Index, which revealed a significant association with symptom severity (CARS: *β* = 0.530, *p* < 0.001; ADOS-SA: *β* = 0.290, *p* = 0.044) even after controlling for screen time. This observed lead-cadmium synergy may explain stronger symptom correlations than single-metal studies, with co-exposure reducing glutamic acid decarboxylase (GAD67) activity by 37%, severely compromising GABA synthesis and exacerbating excitotoxicity (52). Furthermore, the clearance of such toxicants from the brain may be influenced by the glymphatic system, whose dysfunction has been linked to psychiatric disorders and could be a novel pathway modulating metal-related neurotoxicity (53).

Discrepancies across studies may arise from regional exposure differences, biomarker choices (blood vs. hair), and analytical approaches. Our findings, particularly the robust association of the combined metal index, underscore that assessing co-exposure is critical. This supports a two-pronged clinical strategy. First, consider targeted screening for heavy metal exposure in children with ASD, especially those with pronounced social impairments. Second, implement personalized nutritional management to address potential deficiencies that may interact with such toxicants (54).

This study identified significant positive correlations between serum VD levels and gross motor development in children with ASD, consistent with prior research (3, 55, 56). A randomized controlled trial confirmed that vitamin D3 supplementation enhances motor skill acquisition in this population (57). Mechanistically, VD regulates motor development through three primary pathways: (1) vitamin D receptor (VDR)-mediated promotion of myosin synthesis in type II muscle fibers, where deficiency causes hypotonia and delays milestone achievement (prolonging development by 2.3–3.1 months) (55); (2) neuroprotection through the upregulation of brain-derived neurotrophic factor (BDNF) and glutathione to mitigate oxidative damage, coupled with the modulation of dopaminergic pathways to support motor coordination (58–60); and (3) attenuation of neuroinflammation-induced cerebellar-basal ganglia circuit dysfunction that impairs balance and motor imitation (61). Given the established efficacy of VD supplementation in clinical trials (62), routine monitoring of vitamin D status and targeted supplementation to maintain optimal levels is a warranted and concrete component of ASD management.

The relationship between VB12 and neurodevelopment appears more complex and context-dependent. While our data suggest a positive association with motor function, other large studies report null findings for direct correlations, noting instead significant effects in specific subgroups (e.g., children aged 2–4 years) and on different developmental domains (e.g., fine motor and adaptive skills) (3, 63). The metabolic consequences of VB12 deficiency, particularly homocysteine accumulation, suggest the existence of indirect mechanisms that impair motor function (OR for motor coordination deficits = 3.42; 95% CI: 1.87–6.24) (64). These mechanisms involve impaired myelination and oxidative stress, which damage the spinal cord-cerebellar pathways and thereby disrupt motor neuron signaling and synaptic plasticity (63, 65). Therefore, although not universally correlated with gross motor scores, assessing VB12 status remains clinically relevant, particularly in younger children or those with concurrent adaptive or fine motor concerns.

Taken together, our results on VD and VB12, alongside the previously discussed associations involving iron and heavy metals, underscore a central theme: discrete nutritional and environmental factors are linked to specific neurodevelopmental profiles in ASD. This supports a shift toward integrated and personalized assessment strategies in clinical practice. Moving forward, precisely defining which biomarkers to monitor (e.g., VD, iron, heavy metals) and in which clinical subpopulations will be key to translating these associations into effective, individualized interventions.

Beyond nutritional deficiencies, dysregulation of essential trace elements like Cu may also contribute to neurodevelopmental impairments in ASD. Our study adds to emerging evidence of Cu imbalance by demonstrating a significant negative correlation between serum copper levels and gross motor development. This finding aligns with reports of elevated Cu levels in blood and hair samples of children with ASD, particularly in those with more severe symptoms (66, 67). Pathophysiologically, excessive copper may disrupt motor function by interfering with neurotransmitter systems (especially glutamatergic signaling), inducing oxidative stress in motor neurons, and impairing synaptic plasticity critical for motor learning and coordination (68). These mechanisms likely underlie the balance, gait, and motor planning difficulties observed in a substantial subset of children with ASD (69). Therefore, alongside monitoring for nutritional deficiencies, clinical assessment should consider the potential for copper dysregulation, especially in children with significant motor delays. Defining the utility of routine copper screening and understanding its interaction with other metabolic factors remains an important goal for future research.

The role of zinc in our cohort warrants specific comment. While some literature, including meta-analyses, links zinc deficiency to ASD (70, 71), our study did not find significant differences in serum zinc levels between ASD and TD groups, nor significant correlations with core symptoms. This discrepancy may reflect several factors: regional and dietary variations influencing baseline zinc status, differences in ASD sub-phenotypes across studies, or the possibility that zinc dysregulation in ASD is more subtle or occurs at the cellular/tissue level rather than being consistently reflected in systemic circulation. Our findings do not contradict the established biological importance of zinc in neurodevelopment but highlight the heterogeneity of ASD populations and the need to interpret nutrient biomarkers within specific clinical and geographical contexts.

In summary, our study delineates distinct profiles of association: environmental co-exposure to lead and cadmium with social deficits, imbalances in specific nutrients (e.g., vitamin D, copper) with motor domains, and altered levels of vitamins like B12 with more complex, age-dependent outcomes. This pattern underscores that ASD is not associated with a global nutritional deficit, but rather with selective dysregulation of both beneficial and toxic elements, each linking to specific symptom domains. These findings advocate for a multidimensional biochemical assessment in the clinical management of ASD, moving beyond general supplementation toward personalized interventions based on individual biomarker profiles and specific developmental challenges. Future longitudinal studies should aim to establish causal pathways and test the efficacy of such targeted, biomarker-informed strategies.

## 5 Limitations

This study has several limitations that should be considered when interpreting the findings. First, the cross-sectional design precludes the establishment of causal relationships between micronutrient/heavy metal profiles and ASD symptoms or neurodevelopmental outcomes. Second, the moderate sample size may limit the statistical power to detect smaller effects and the generalizability of the results. Third, the selection of specific vitamins, minerals, and heavy metals was based on their established biological relevance and prior associations in the ASD literature, rather than an unbiased omics approach. This targeted panel may have omitted other potentially relevant biomarkers. Fourth, we lacked detailed quantitative data on dietary intake and broader environmental exposures, which are potential confounders or effect modifiers. Fifth, although data on maternal parity, caregiver age, and education level were collected, these variables were not included as covariates in the primary regression models—a decision made to preserve statistical power and focus on direct biological relationships; however, these and other unmeasured sociodemographic factors represent potential sources of unmeasured confounding and reflect the inherent heterogeneity of the ASD population. Sixth, given the exploratory nature of correlating multiple biomarkers with several clinical outcomes, the possibility of Type I error (false positives) due to multiple comparisons should be considered when interpreting the results. Future studies with larger sample sizes, detailed dietary assessments, unbiased biomarker discovery, and multicenter designs are needed to confirm these preliminary findings and to better account for potential confounders.

## 6 Conclusion

This cross-sectional study identified significant associations between micronutrient and mineral profiles and clinical manifestations in children with ASD. Notably, co-exposure to lead and cadmium, as reflected by a combined exposure index, was independently associated with more severe social communication impairments, even after accounting for screen time. These findings underscore the potential clinical relevance of integrated biomonitoring, which encompasses both nutritional status and environmental heavy metal exposure. However, this interpretation requires caution in light of the study’s limitations, notably its observational design and moderate sample size. Looking forward, our results highlight the value of considering multifaceted strategies that address individualized nutritional imbalances and mitigate environmental risk factors within comprehensive ASD care models. Future longitudinal research is needed to confirm these associations and to evaluate the efficacy of such targeted monitoring and intervention approaches.

## Data Availability

The original contributions presented in the study are included in the article/supplementary material, further inquiries can be directed to the corresponding author.
